# A Mini-Review Regarding the Modalities to Study Neurodevelopmental Disorders-Like Impairments in Zebrafish—Focussing on Neurobehavioural and Psychological Responses

**DOI:** 10.3390/brainsci12091147

**Published:** 2022-08-28

**Authors:** Alexandrina S. Curpăn, Ioana-Miruna Balmus, Romeo P. Dobrin, Alin Ciobica, Gabriela E. Chele, Dragos Lucian Gorgan, Alexandra Boloș

**Affiliations:** 1 Department of Biology, Faculty of Biology, “Alexandru Ioan Cuza” University of Iasi, Bd. Carol I, 20A, 700505 Iasi, Romania; 2 Department of Exact Sciences and Natural Sciences, Institute of Interdisciplinary Research, “Alexandru Ioan Cuza” University of Iasi, Alexandru Lapusneanu Street, No. 26, 700057 Iasi, Romania; 3 Department of Psychiatry, Grigore T. Popa University of Medicine and Pharmacy, 700115 Iasi, Romania

**Keywords:** zebrafish, neurodevelopmental disorders, behavioral test, morphology

## Abstract

Neurodevelopmental disorders (NDDs) are complex disorders which can be associated with many comorbidities and exhibit multifactorial-dependent phenotypes. An important characteristic is represented by the early onset of the symptoms, during childhood or young adulthood, with a great impact on the socio-cognitive functioning of the affected individuals. Thus, the aim of our review is to describe and to argue the necessity of early developmental stages zebrafish models, focusing on NDDs, especially autism spectrum disorders (ASD) and also on schizophrenia. The utility of the animal models in NDDs or schizophrenia research remains quite controversial. Relevant discussions can be opened regarding the specific characteristics of the animal models and the relationship with the etiologies, physiopathology, and development of these disorders. The zebrafish models behaviors displayed as early as during the pre-hatching embryo stage (locomotor activity prone to repetitive behavior), and post-hatching embryo stage, such as memory, perception, affective-like, and social behaviors can be relevant in ASD and schizophrenia research. The neurophysiological processes impaired in both ASD and schizophrenia are generally highly conserved across all vertebrates. However, the relatively late individual development and conscious social behavior exhibited later in the larval stage are some of the most important limitations of these model animal species.

## 1. Introduction

The latest edition of the Diagnostic and Statistical Manual (DSM) [1] defined neurodevelopmental disorders (NDDs) as neurological diseases impairing all the cognitive functions and, by consequence, impacting attention, intellectual performances, social behavior, and communication. An adequate diagnostic of a mental disorder involves multifactorial approaches, including social, psychological, and biological factors, as highlighted in the fifth edition of the DSM. An important feature of all the NDDs is the onset of the symptomatology early during childhood and their co-occurrence is quite common [2]. The classification of the NDDs in eight classes, depending on the cognitive function which is mainly impaired, emphasized the multifactorial etiopathogeny of these disorders, represented by intellectual, communication, autism spectrum disorder (ASD), attention-deficit/hyperactivity disorder (ADHD), specific learning, motor, tic disorders, and others [1,2]. Cognitive impairment is a common feature for all of these disorders, which are included in one diagnostic category and thus involves the complex biopsychosocial context. 

The etiopathogeny of the NDDs can be discussed using two main approaches, neuropsychological account and neuroconstructivism. The neuropsychological account represents the module-based organization of the physiological brain. The neuroconstructivism account better describes the pathological mechanism of NDDs and states that the brain is a self-organizing neuronal network that fitly interacts to produce physiologically active subsystems [3]. The symptoms of all the NDDs are sometimes overlapped, and lead to challenging clinical diagnosis [4]. While it was not traditionally defined as a NDD and its NDD-similar traits were extremely argued, the etiopathogeny of schizophrenia was also described indirectly using both of these approaches [5,6,7].

Thus, the clinical studies and the studies performed on animal models can reveal some important characteristics of the etiologic and pathologic mechanisms of NDDs. Animal models are nowadays an important tool for biomedical research. Among all of these animal models, the zebrafish (*Danio rerio*) model is currently preferred for most studies, such as those in genetics, neurophysiology, biomedicine, toxicology, and developmental biological research. This model species has numerous advantages regarding the living and breeding conditions, central nervous system morphology, genetics [8,9], specific fertilization and ex-utero embryonic development [10,11], which are relevant for NDDs research. Vaz et al. [11] thoroughly presented the potential of NDDs teleost models and suggested that the new studies can benefit from the zebrafish physiological and genetic background to combine components of the often-multifactorial NDDs for further targeted models, mechanisms, and personalized treatments. 

On the one hand, ASD are severe mental disorders involving the early impairment of cognitive and socio-affective development. On the other hand, schizophrenia occurs as a consequence to certain genetic and in utero contexts generating specific phenotypical anatomo-physiological traits associated with pathological development, often found similar with NDDs in terms of time onsets [12]. Animal models could provide a better understanding of these disorders’ pathophysiological mechanisms. Despite that the mammal models are currently preferred, some studies suggested that a less complex animal model, such as the zebrafish and other teleosts models, could issue a more relevant research context [13]. 

The aim of our review is to underline the evidences regarding the utility of vertebrate animal models for NDDs and schizophrenia research and to specifically highlight the relationship between the zebrafish neurodevelopmental aspects and the ASD and also schizophrenia-relevant abnormalities in development, anatomy, and behavior. 

## 2. Methods

A literature search was performed on the main available scientific databases (PubMed/Medline, Google Scholar, Embase, Web of Science, Scopus, Science Direct). All scientific articles written in English language and published after January 2000 were screened using keywords, such as (but not restricted to) “neurodevelopmental disorders”, “zebrafish”, “embryo”, “larvae”, “schizophrenia”, “autism”, “neurobehavioral development” and their combinations. Exclusion criteria consisted of the publication in a language other than English or in other time intervals of NDDs animal model studies other than ASD or schizophrenia models. All the data was collected by two separate investigators. Any differences in opinion regarding the characteristics of the included studies were solved by common consent and/or supplemental screening carried out by a third investigator.

## 3. Pathophysiology and Mechanisms

The onset of the majority of the disorders included in the category of NDDs is during the childhood period, but the specific mechanism or the nature of the onset is still controverted. In a recent study regarding the pathophysiological development patterns of ASD, Ozonoff et al. [14] observed gap between the traditionally conceptualized onset of the symptoms and the cognitive patterns of the ASD onset in children. Also, the traditional definition of ASD highlight neither the correlation between the visible clinical manifestation and the molecular background, nor the predominant genetic component of this disorder. Thus, since some pathological patterns produced similar clinical symptomatology [15], many ASD etiological theories were not confirmed. 

By the age of two, most of the ASD clinical manifestations could easily be observed: important debility in social behavior, communication difficulties (or the absence of verbal or non-verbal response to social stimulation), and restrictive or repetitive behavior [16]. However, during the first year of life, despite that many behavioral and cognitive symptoms already occur, they are often missed in the early diagnosis being less visible [14]. Thus, the specific ASD clinical manifestations are preceded by the less clinically visible pathological mechanisms that determine these symptoms. 

Many recent studies underline that neuronal mitochondrial dysfunction was frequently correlated with neurodevelopmental delay (learning and speech retardation), hyperactivity, abnormal social interaction, limited interests, stereotypical and repetitive behavior, seizures, and self-destructive behavior [3,16]. Also, the excessive production and accumulation of mitochondria-originating reactive oxygen species can trigger molecular response cascades affecting enzymatic modulation, membranes permeability and fluidity, ions dyshomeostasis, and apoptotic signaling. 

Despite that schizophrenia could not be considered a traditional NDD, some pathophysiological traits could suggest that this disease can be described as an atypical NDD [17,18]. For example, schizophrenia development is partly based on the N-methyl-D-aspartate (NMDA) receptor potential to exhibit an excitatory effect on the glutamatergic neurons. This hypothesis is built on the previous observation that excitatory neurotransmission dysfunction could contribute to the neuropathological development of schizophrenia [19]. However, the NMDA receptors inactivation leads to impaired socio-affective behavior, delayed learning, but normal sensitivity to feedback, as seen in the traditional NDDs (a mice model of inactivation of NMDA receptors on dopaminergic neurons) [20], while the loss of NMDA receptive neurons via autoimmune reaction initially mimics schizophrenia symptomatology (despite that this mechanism was not previously described in schizophrenic patients) [21]. In this context, many of the pharmacological solutions for schizophrenia management are based on the NMDA receptors deficiency (as seen in the traditional NDDs) rescue which can be successfully achieved in the adult brain together with schizophrenia-associated symptomatology remission [22,23]. 

However, the multifactorial pattern of schizophrenia also includes the aberrant function of fast-spiking parvalbumin-positive interneurons (PVIs) which leads to excitatory imbalance. PVIs together with myelination defects may be responsible for schizophrenia’s pathophysiology aspects and symptoms in early adulthood. Defects in PVIs and their associated networks might be caused by the interaction of two interdependent factors: brain redox imbalance and NMDA receptor (NMDAR) hypofunction [24]. On the other hand, a parvalbumin-related pathophysiological hypothesis was described for ASDs. Besides the ASD shared schizophrenia-related parvalbumin neurons impairment pathway, this hypothesis also suggested that no less than four other pathophysiologic changes could lead to different ASD specific pathologic traits development, including calcium-voltage channels corruption due to Ca^2+^ supply extracellular misplacement (also leding to GABA release) [25].

Furthermore, the Purkinje cells’ dendritic field morphological changes, such as the loss of distal and terminal dendritic branches, and the dendrites length reduction were also observed and described in schizophrenic brains [26]. 

## 4. Zebrafish Physiology, Neurodevelopment, and Behavior

The small and well-adapted to controlled environment zebrafish is currently preferred for biomedical research and offers the opportunity for various applications [10]. The fully characterized zebrafish behavior as well as the significantly similar genome provides sufficient background for its fitness in NDDs and schizophrenia research. 

Despite the general belief that less complex animals exhibit fewer complex features, it was previously shown that zebrafish behavior consists of most of the cognitive and socio-affective traits, closely resembling or equivalent to human behavior. Moreover, this highly social species start manifesting some of the social behaviors relevant to ASD and schizophrenia as early as the embryo stage, in a measurable manner. The zebrafish behavioral assessment includes the evaluation of many visual, acoustic, tactile, and olfactory stimuli responses. Although it is considered to be less complex than the mammal models and human, it was showed that zebrafish neurobehavioral response to stimuli is similar to humans, while its modulation could specifically resemble some of the schizoaffective impairments with equivalent neurodevelopmental descent (Table 1). For an instance, ASD patients exhibit limbic system-driven hypersensibility in neurobehavioral response to olfactory stimuli [27]. In zebrafish models, the olfactory responses to chemical stimuli was characterized by anxious behaviors suggesting that the basic amygdala-prefrontal cortex-like circuits. However, they are not modulated by specific pathways, or by prefrontal cortical structures. Despite that, they could exhibit neurobehavioral responses comparable to human [28,29]. In a schizophrenia zebrafish model (nrg1 genetic model), the impaired response to olfactory stimuli was correlated with decreased volumes of the olfactory bulbs [30], this feature and the olfactory bulb afferents-receiving brain areas with reduced cortical volume being also seen in schizophrenic patients [31]. In accordance with these aspects, since stimuli perception triggers memory processes, it was showed that short-term, long-term, and discriminative memory concur with complex behaviors, such as anxiety/fear, social, reward-related, sexual, pain-related, sensory, and behavioral patterns associated with sleep [32,33,34], all being important in the NDDs context. 

Zebrafish exhibit specific physiological traits (translucent body) and ontogenetic development (embryo, eleutheroembryo, larvae, and juvenile) that provide advantageous cellular types, tissues discrimination, and optogenetic manipulation [51,59]. Gills and skin permeability can be observed at 72 hpf (hours post-fertilization) [60,61], while the activity of blood-brain barrier was demonstrated as early as at 36 hpf [62] which means that chemical induction of ASD and schizophrenia-like impairments could be performed in the early stages of the neuronal circuitry development. This could support neuronal system evaluation during the first stage neurogenesis, a rather difficult task in rodent models [63]. Moreover, these specific traits enable the possibility to modulate and study the central nervous system development with regards to NDD-affected mechanisms in a less complex and shorter time spanned neurodevelopmental context provided by the zebrafish brain. 

Also, some of the brain structures susceptible to neurodevelopmental impairments or prone to be affected by the NDDs are similar to human. The main brain structures of zebrafish with similar cell types are the hippocampus (anterodorsolateral pallium), the diencephalon, tectum, tegmentum, and the cerebellum [6]. 

Many scientists argue the use of zebrafish as models for some complex mental disorders which are characterized by the alteration of the prefrontal cortex and expanded telencephalon functions [64], as the zebrafish lack these structures. Despite that, their functions are partly taken over by homologous structures [8,51,65]. Moreover, despite of not possessing an expanded telencephalon, many of the vertebrates can exhibit complex cognition and can be the subject of decision-making behavioral tasks [64]. Evidence supporting this statement was brought by Terigoe and colab. Ref. [66] in a study published in Nature Communications describing the neural activity of adult zebrafish facing decision-making tasks in response to colored visual stimulation and aversive stimulation learning. Thus, it was showed that the dorsal pallium, the evolutionary equivalent to mammalian striatum and globus pallidus, could be implicated in the virtual visual stimuli perception and response suggesting a rudimentary internal model of the outer cognitive world (seen in other higher vertebrates). Moreover, the Terrigoe et al. [66] study reported clusters of neurons apparently originating in the visual stimuli perception areas of the zebrafish brain that were reactivated during the cognitive rule assignment and prediction error. Similar processes were identified in zebrafish larvae, as Bahl et al. [67] reported three neuronal clusters from the larvae anterior hindbrain associated with individual behavioral choices. The later presented an innovative technique to evaluate decision-making behavior in zebrafish using virtual reality and translation to reality training, being a step further in artificial intelligence use to assess behavioral patterns followed by machine learning and prediction as hypothesized by Xu et al. [68]. 

Moreover, in Balh et al. [69] study the neural circuits changes were evaluated using electrode-based imaging and calcium signaling. Calcium imaging of the zebrafish brain is yet another innovative technique assessing neural activity, recently described in a study regarding the cerebellar radial glia developmental patterns in zebrafish larvae by correlation to morphodynamic changes [69]. Furthermore, Zylberthal and Bianco [70] reported the calcium imaging technique combined with mechanistic network modelling in zebrafish larvae to evaluate spatial and temporal network interactions in the zebrafish optic tectum in response to visual stimuli (prey-catching responses). 

Another key feature that makes zebrafish extremely valuable in research and in NDDs study, particularly, is that the complete development of the zebrafish brain, the development of the nervous system, and the maximum cognitive potential reached within the first 90 days of life [7]. While the complete life cycle unfolds on 4 to 5 years, the pre-adult stages are fastly progressing with short-termed steps: pre-hatching embryo (0–72 h post fertilization), post-hatching embryo (72–120 hpf), larvae (5–29 dpf, days post fertilization, free swimming), and juvenile fish (30–89 dpf) [6]. Kimmel et al. [58] demonstrated that the neurogenesis starts at 16 hpf (pharyngula stage of the zygote, primary neurogenesis) and lasts until 72 hpf (early larvae stage, secondary neurogenesis). Thus, by the end of embryonic gastrulation (10 hpf), the central nervous system precursors form the neural plate, neural tube, expanded brain, and spinal cord. Some of the primary neurons formed after 24 h post-fertilization mediate early escape reflex behavior (coordinated larval behavior is provided by the simple neuronal networks) [71]. This particular behavior gains relevance in ASD and schizophrenia zebrafish models as both diseases are associated with stimuli response impairments. The escape reflex behavior is mediated by hindbrain pathways which mean that the observed changes could be associated with ASD-like impairments, since the main brain structure affected in ASD is the cerebellum [8]. Moreover, the forebrain, the hindbrain, and the limbic system were often described as the most damaged brain structures in schizophrenia [72].

During hatching, the second neurogenesis stage (2–3 dpf) and the more complex movement and behaviors needed for survival are occurring. Thus, the primary circuitry is replaced by the second wave neurogenesis, when the components of each segment of the main central nervous system (spinal cord, hindbrain, midbrain, and forebrain) differentiate and gain function [29,71]. These aspects could be vital in NDDs and schizophrenia research since it was showed that both ASD and schizophrenia neurodevelopmental changes are occurring in the early stages of brain structures differentiation and specialization [73] leading to the first prodromal signs, such as motor delays, atypical visual orientation, and aberrant response to social stimulation in the case of ASD, while the neurodevelopmental hypothesis of schizophrenia pathophysiology supports the altered patterns of brain growth—ventricular enlargement, temporal lobes reduction, and abnormal septum pellucidum [74].

The zebrafish has the capacity to exhibit quantifiable behavioral responses to external stimuli, early during the developmental stages. The muscular movements (spontaneous coiling movements), as early as 17 hpf, are the result of non-brain-controlled automatisms believed to be implicated in the hatching process occurring at 3 dpf. However, escape response-type swimming behavior could be observed in untimely dechorionated embryos (2 dpf or even earlier), as the brain-controlled movements response to external stimuli could be observable stating with 2 dpf [8]. Thus, even from early larva stage, any brain impairments associated with aberrant moving patterns can be evaluated. Many innovative behavioral assessment tools are currently available to use in embryo and larvae models studies. The behavioral analysis of juveniles and adults is more facile and is carried out using water environment adapted mazes (similar to rodent mazes) and video capturing systems assisted by zebrafish behavior analysis software (such as EthoVision or DanioScope from Noldus, or the newer BonZeb [75]), but embryo and larvae behavior assessment is much more delicate due to the specific traits of these life stages (reduced dimension, discrete behavioral patterns and parameters). Thus, highly sensitive logistics are required, one of which being the ZebraBox developed by ViewPoint Behavior Technology (Lyon, France). ZebraBox provides extremely accurate tools for zebrafish larvae behavioral assessment and was recently used to evaluate sleep patterns [76], locomotor activity [77,78], social behavior [78], and behavioral effects of maternal exposure to chemicals [79]. Moreover, it was successfully used to assess autistic-like behavior in zebrafish larvae lacking an important neurodevelopmental factor, nde1 [80], and to evaluate a difficult way to model mild traumatic brain disorder in zebrafish larvae using rapidly decelerating linear movements [81]. ZebraLab, MicroZebraLab, and VisioBox (ViewPoint Behavior Technology, Lyon, France) could also be used in zebrafish embryo and larvae behavioral and morphological assessment. 

In this way, the acoustic startle reflex (C-start) is one of the earliest behavioral tasks administered to zebrafish larvae. Also, learning and habituation are occurred in the fast-developing zebrafish embryos and larvae. Thus, the post-synaptic signaling determined by the sound stimuli could be recorded as early as 3 dpf in zebrafish embryos and starting with 4 dpf in zebrafish larvae. Roberts et al. [82] demonstrated that rapid escape reflex response to 50–1000 Hz acoustic startling stimuli could be repeatedly and persistently observed in zebrafish larvae older than 5–6 dpf. The physiology of startle response includes the Mauthner cells stimulation, a pair of bilateral reticulospinal neurons localized in the caudal hindbrain [83] and modulated by the ipsilateral acoustic neurons from the mechanosensory hair cells. In humans, the anxiety and autistic symptoms are correlated with hypersensitivity, which is induced by low threshold of acoustic startle response [82]. Also, habituation-related sudden involuntary motor reactions could be modulated by weaker non-startling stimuli, as observed in Huntington’s disease, and schizophrenia [32,84]. The NMDA receptors can successfully modulate the startle reflexes and habituation [85] thus suggesting a clear correlation between the quantifiable zebrafish behavior and the pathophysiological trait of ASD in human. Another aspect relevant for ASD is represented by the NMDAR-modulated rapid habituation behaviors in zebrafish larvae, the transgenic mice studies demonstrated that early GABAergic signaling correction can prevent autistic-like behaviors during later development [86,87]. 

**Figure 1:**
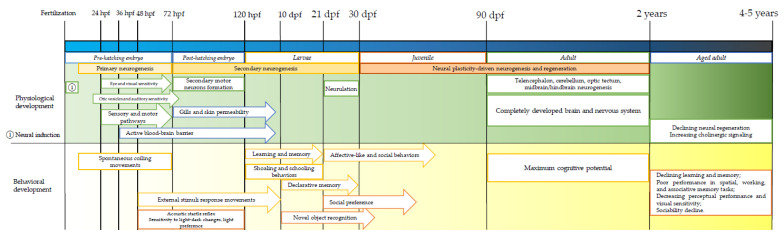
Neurophysiological and behavioral development of zebrafish [54,89,90,91].

The light-dark test was also showed to retrieve good quality results in zebrafish behavioral analysis with regards to the pathophysiology of ASD and schizophrenia, based on NMDAR modulation. Normal behavioral patterns for zebrafish are represented by the preference for dark areas for adult specimens, while the larvae preferred the light areas [88]. Kalueff et al. [88] observed that anxious-like behaviors (freezing and diving) and social preference are modulated by NMDAR. These behaviors are developed at the age of three weeks. The first social behavior is occurring in the post-embryonic stage of the zebrafish and can be identified in the larvae tendency to form groups—shoaling behavior [54]. Also, shoaling behavior gains complexity between 6 and 21 dpf due to the development of rudimentary social preference for conspecifics, based on visual stimuli [56]. Contrary to previous studies, Meshalkina et al. [8] suggested that social preference test can be a reliable sociability evaluation in larval stage (Figure 1), while Engeszer et al. [54] demonstrated that shoaling preferences develop as late as in the juvenile stage.

In ASD, but also in schizophrenia, significant impairments of the declarative memory can be noted. Also, the abnormal brain lateralization observed in both pathologies suggests that memory recollection and emotionally processing are impaired [92]. In zebrafish, as well as in other animal model species, declarative memory performance evaluation could be performed using the novel object recognition. Thus, it was shown that 10 dpf zebrafish larvae could successfully process and recollect the presence of novel object in their swimming tank using both short-term and long-term memory [93]. Moreover, the left eye system (preferred in zebrafish novel object recognition interpretation) was showed to be active in 8 dpf zebrafish larvae. This suggests that cognitive development of zebrafish larvae as early as this provides sufficient complexity to appraising novelty [94]. There are many controversies regarding the involvement of the short versus long-term memory in novel object preference, although many new studies on object recognition acknowledged zebrafish ability to recollect memories. May et al. [95] demonstrated the preference for the familial object (previously exposed to the object for less than 10 min). Also, Lucon-Xiccato and Dadda [96], Magyary et al. [97] clearly pointed out the preference of the zebrafish for the solid medium-sized and floating novel objects. However, there is no strong evidence to argue the preference of the zebrafish for novel object, except for the study methodology and the time-framed object positioning. 

## 5. Autism and Schizophrenia Zebrafish Animal Models

Many psychiatric disorders occurring in human were not described in most of animal species. Thus, the aim of animal modeling in psychiatric research consisted in obtaining specific symptoms of the disorders and/or mechanistical simulation [98]. Many studies highlight that non-human primates and other mammal species could naturally exhibit ASD-like behavioral traits, such as changes in sociability and attention [99]. For example, canine dysfunctional behavior is a rare animal behavioral disorder which closely resembles the human profile of ASD. Moon-Fanelli et al. [100] demonstrated that male dogs are predominantly prone to develop repetitive behavior (obsessing tail-chasing, chewing), aberrant sensory and social stimuli response, and impaired affective and social behavior (phobias, aggressivity, social isolation) in relation to intraspecific interaction or in relation with human. A recent study published by the Royal Society of Open Science suggested that the social and cognitive aspects of the neurobehavioral status of dogs are similar to the behavior of human infants [101].

Thus, Falcao et al. [102] presented the suitability of zebrafish embryos in toxicological research by describing the characteristics of the the acute (12 h) and chronic (24 h) exposure to different agents. Also, they reported measurable changes in locomotor activity, visual motor response [89], and neurodevelopmental defects suggesting that chemically-induced zebrafish embryo models could be used in ASD and schizophrenia research with yielding comparable to rodent models. 

Besides the typical ways to induce ASD and schizophrenia-like behavioral impairments, several studies reported that that methyl-mercury II chloride [103] and gold [104] administration could lead to changes in cognition and socio-affective behavior of zebrafish. Strugaru et al. [103] showed that the acute exposure methyl-mercury II chloride could lead to decreased locomotor activity, impaired decision-making behaviour (fewer full clockwise rotation events, incapacity to recollect the association between colours and positive/negative stimuli), anxiety-like behaviour (freezing events), and impaired social behaviour (decreased preference for social interaction, decreased aggressivity). The same group showed that the acute exposure to gold could result in similar toxic neurobehavioral effects, such as decreased locomotor activity, impaired decision-making behaviour, and decreased sociability, in a reversible manner [104]. These studies suggested that the exposure to heavy metals could induce significantly NDDs-associated behaviours. Yet it is not clear how the heavy metals exposure could determine the pathophysiological processes undergone in ASD and schizophrenia. However, Frye et al. [105] suggested that the exposure to heavy metals during the early stages of life could be interfering with the mitochondrial metabolism and the neurodevelopment of the foetus in human. Due to the possible controverted intervention in the in utero development, cautions about the exposure to heavy metals and many potentially harmful compounds (such as pesticides) have been formulated [106,107]. 

Similarly, controverted results regarding the possible neurodevelopmental effects on the foetuses and the predisposition to psychiatric impairments in offsprings, such as hyperactivity-inattention disorder, conduct-oppositional disorders, and other [108] were reported for caffeine consumption during pregnancy. Patti and colab. repeatedly reported the possible vulnerability to ASD-related behaviours in children descending from caffeine consuming mothers [109,110]. Moreover, Christensen et al. [111] described several significant structural brain alterations and deleterious neurocognitive outcomes in children as being associated with caffeine consumption in pregnant women. Since the correlated changes were observed for the left corticospinal tract and left inferior fronto-occipital fasciculus (decreased neuronal density), it could be suggested that the gestational caffeine exposure interfered with the neurodevelopmental processes occurring in brain areas associated with working memory formation and visual stimuli perception [111]. Also, some animal studies showed that gestational caffeine exposure could lead to hippocampal hyperexcitability and GABA neurons migration impairments [112]. 

Due to their transparent appearance, several studies suggested that the zebrafish embryos mutated models can be used for the evaluation of the specific gastrointestinal impairments in ASD (see Table 2). James et al. [113] presented the endocrine/neural-dependent glutamatergic regulatory mechanisms of the digestive motility in zebrafish embryos, which is very similar with the human equivalent mechanism. Also, they observed the potential of human SHANK3 isoform to rescue digestive activity in impaired embryos suggesting the utility of different developmental stages vertebrate models for the research of NDDs pathological mechanisms. 

The genetic components of the NDDs studied using the ASD and schizophrenia zebrafish models mostly comprise in knock out/knock down mutants or transgenic models. The microcephaly seen in the brain of patients with schizophrenia, and macrocephaly seen in ASD brains are in relationship with ktcd13 overexpression and knock down, as reported in some zebrafish models [8,11].

Several studies evaluated the relationship between the specific pathways of ASD and schizophrenia, the neurodevelopmental mechanisms, and behavioral patterns of the different development stages of zebrafish. Thus, genetic models, such as cntnap2 knock down model (relevant to ASD seizures), nbea mutant model (GABAergic synaptic plasticity impairments, impaired locomotor activity, and decreased startle reflex), and DIA1/DIA1R transgenic models (cognitive retardation) were successfully described [8,11]. Also, the neurodevelopmental impairments in schizophrenia, such as cerebellar neurons loss of function, demyelination, and oligodendrocytes development defects can be studied using disc1, nrg1, and cacna1d mutant zebrafish larvae [114]. The newer model of Xie et al. [115] studied the implication of pdzk1 gene in both schizophrenia and ASD, the knock-out individuals showing increased spatial frequency tuning functions, thus abnormal visual behavior. 

Regarding the potential to generate new techniques using the recently unravelled genetic tools, a novel in vivo spatiotemporal activation of specific cell signaling was described by Moro et al. [116]. Reporter and fluorescent protein genes were used to generate extremely specific fluorescent markers. Microinjected in the embryos, the transgenic biosensor could reveal neural activity and development from embryo to juvenile life stages.

**Table 2:** Toxicological and phenotypical studies conducted on zebrafish models.

Study	Modulatory Agent	Stage of Development	Observed Effects in Zebrafish	Interspecific Translation
[102,117]	Valproic acid	Embryos	↓ Locomotor activity; ↑ Locomotor activity (intraperitoneal administration); Dose-depend response	In valproic acid in utero exposed rats [118,119]: ↓ Locomotion; ↓ Ultrasonic vocalisation (perception and response); ↓ Social preference and interaction; Cognitive rigidity; Repetitive behaviour.
[120,121]	Valproic acid	Embryos/larvae	Telencephalon: neural progenitor cell proliferation in the mutagenesis: adsl, mdbs, tsclb, shank3	
[122]	Valproic acid	Juveniles	↑ Locomotor activity; ASD-like social behavior.	
[6,123,124]	Valproic acid	Embryos, larvae	Growth retardation and congenital abnormalities (embryos); Behavioural impairment—social interaction deficits; ↓ Locomotor activity.	In rodents: similar developmental and behavioural outcomes
[6,93,125,126,127,128,129]	MK-801 (NMDAR antagonist)	Larvae	Disrupted memory formation (T-maze); ASD-like behaviour; Shoaling cohesion: locomotor actions are dose-dependent (response at 5 dpf); Disrupted glutamatergic pathways necessary for PPI (schizophrenia phenotype).	Disrupted memory retrieval (cognitive set-shifting); Locomotor responses as early as day 3 postnatal; ↑ Locomotor activity; Social withdrawal.
[113,129,130,131]	Shank3a/shank3b mutated model	Embryos	ASD-typical behavior and digestive impairments	In juvenile Shank3 deficient rats: social communication deficits
[132,133,134,135]	CHD8 mutated models	Larvae	Reduction in postmitotic enteric neurons leading to impaired gastrointestinal motility Macrocephaly	In transgenic mice: Gastrointestinal impairments associated with ASD; Macrocephaly.
[136,137,138]	Chd7 null mutant models	Larvae	Reduced vagal and enteric innervation, decreased intestinal motility; GABAergic defects, hyperactivity.	In mice: cerebellar hypoplasia
[139,140]	Kctd13 human mRNA injection in embryos; Kctd13 mutants	Embryos/larvae	Overexpression: microcephaly; Knock down: macrocephaly.	In mice: impaired synaptic transmission
[102,117]	Caffeine	Embryos	↓ Locomotor activity; ↑ Locomotor activity (when adm. intraperitoneal); Dose-dependent response.	Adults rats: ↓ Locomotion
[22,34]	Nicotine	Embryos (46 hpf)	Hyperactivity at high doses	Rodents: hyperactivity
[102,141]	*Eclipta prostrata,* *Spilanthes acmella* (Linn.) Murr	Embryos	Delayed pigmentation development; Incomplete development of the tail and eyes at 1% extract administration; No hatching observed at 40% extract administration.	
[142]	*Polygonum multiflorum*	Embryos	Morphological defects were observed from 105 mg/L	
[102,142]	*Millettia pachycarpa*	Embryos (blastula stage)	↑ Occurrence of spinal curvature; ↓ Heart beat rate; Insensitivity to touching.	
[102,136]	Celastrol	Embryos	At 0.5 µM—developmental abnormalities; At 1.0 µM—lower hatching rates; At >2.0 µM—death.	
[102,143]	Arecoline	Embryos	Smaller body lengths; Pericardial oedema, axial-tail curvature; Growth-retardation; Dose-dependent response.	In mice: Increased resorption; Reduced foetal body weight; Decreased embryo viability.
[36,102]	Cannabinoid	Embryos	↑ Locomotor activity (at low conc.); ↓ Locomotor activity (at high conc.).	Similar effects on locomotor activity, but also may be harmful to the development of the foetus and behaviour of the mother.

## 6. Conclusions

NDDs are multifactorial disorders with complex phenotypes and comorbid traits that have not been fully described to date. The zebrafish embryos/larvae demonstrate several key advantages in traditional NDDs research and also in schizophrenia (recently considered a NDD with atypical traits), such as fast development, early observable behavioral responses, and drug-mediated cognitive changes. Thus, pre-adult zebrafish models could be considered eligible ASD and/or schizophrenia animal models. The recent zebrafish models studies showed that most of the main human pathological manifestations can be modulated in zebrafish during their entire development (embryos, larvae, juvenile, adults), despite that they have a less complex physiology. However, these animal models have several limitations, such as the relatively late development of individual or conscious social behavior.

## Figures and Tables

**Table 1:** Similarities and differences between zebrafish and mammals/humans.

	Similarities	Differences/ Particularities	Measurable Behaviors	Clinical Relevance		Ref.
				ASD	Schizophrenia	
Olfactory stimuli response	Olfactory neurons project on telencephalon and diencephalon; Basic amygdala-prefrontal cortex-like circuits; Pathophysiological changes of the olfactory bulb and projection areas.	Odorant receptors projection—non-specific (glomerulus); Olfactory stimuli response not modulated by prefrontal cortical structures.	Chemical or socially driven olfactory response	Hypersensibility to olfactory stimuli	Circuitry desensibilisation of response to olfactory stimuli	[27,28,29,35,36,37]
Visual stimuli response	Stimuli—driven eye orientation (visual processing) is highly conserved in all vertebrates; Motion perception—similar in zebrafish larvae and adults; Posterior brain regions—modulated visual stimuli perception.	Spectral information spreads in all regions of the CNS in zebrafish larvae exposed to coloured stimuli; Zebrafish discriminate colour, shape, size, and orientation of objects.	Visual stimuli response to dark/light changes, to color, and to novel objects in embryos; Target-directed behavior (tracking) starting with 5 dpf (associated with decision-making behavior).	Atypical brain activation in visual detection tasks (focus to local detail, such as contrast and color)	Delays in visual perception, visual distortions	[35,36,37,38,39,40,41,42,43]
Descending motor and premotor pathways	Preserved descending motor and premotor pathways, such as reticulospinal tract, projections from the midbrain and cerebellum to brainstem targets		Locomotor activity	Behavioral hallmarks—repetitive movement and impaired motor response to external stimuli	Increased motor activity, motor glitches (tics, stereotypies), anxiety-related locomotor behaviour changes	[44,45,46]
Spatial memory	Spatial memory pathways involving lateral pallium, similar to mammals	Lateral pallium homologous hippocampus (medial pallium-derived)	Short-term and long-term learning and memory	Episodic memory and working memory deficits	Memory deficits and decreased cognitive performance	[47,48,49,50]
Neurogenesis and synapse plasticity	Neuronal cell type differentiation modulated by both neurogenic genes and proneural genes	Teleost-like primary neurogenesis not described in mammals; Telencephalon formed by eversion in anamniotes and by evagination in amniotes.	Conditioned place preference, dark/light transition, social decision making, aggressivity	Specific changes in learning and memory formation		[6,49,50,51]
				Related to stimuli response	Related to perceiving fear	
Neuromodulatory pathways	All key neuromodulator systems are highly preserved amongst vertebrates		Inhibitory avoidance, e-flat mirror test, perseverative behaviour	Limbic system impairments		[6,49,52,53]
Neuroendocrine modulation	Stress response mediated by cortisol, hypothalamo-pituitary hormones cascade reaction; Stress response pathways striking similar to human.					
Affective behaviour	Modulated by amygdala and habenula (a group of nuclei in the epithalamus)	More complex neurotransmitter-modulated affective display				
Social behavior and sociability	Robust sociability, cortisol-mediated stress (unlike rodent models); Isotocin-mediated social response; coordinated movement—a sign of sociability in juveniles.	More complex hormonal patterns in stress response in mammals	Sociability behaviors starting with embryo movement	Typical social behavior phenotypes		[54,55,56,57,58]

## Data Availability

All date is available based on a simple request to the authors.
